# A Novel Biocompatible Titanium–Gadolinium Quantum Dot as a Bacterial Detecting Agent with High Antibacterial Activity

**DOI:** 10.3390/nano10040778

**Published:** 2020-04-17

**Authors:** Vishma Pratap Sur, Aninda Mazumdar, Amirmansoor Ashrafi, Atripan Mukherjee, Vedran Milosavljevic, Hana Michalkova, Pavel Kopel, Lukáš Richtera, Amitava Moulick

**Affiliations:** 1 Department of Chemistry and Biochemistry, Faculty of AgriSciences, Mendel University in Brno, Zemedelska 1/1665, CZ-61300 Brno, Czech Republic; xmazumda@mendelu.cz (A.M.); amirmansoor.ashrafi@mendelu.cz (A.A.); atripan@gmail.com (A.M.); grizlidripac@gmail.com (V.M.); hanabuchtelova@gmail.com (H.M.); oliver@centrum.cz (L.R.); 2 Central European Institute of Technology (CEITEC), Brno University of Technology, Purkynova 123, CZ-62100 Brno-Královo Pole, Czech Republic; 3 Department of Inorganic Chemistry, Faculty of Science, Palacky University, 17. listopadu 12, CZ-771 46 Olomouc, Czech Republic; paulko@centrum.cz

**Keywords:** bacterial resistance, titanium–gadolinium quantum dots, bacterial detection, antibacterial activity, SECM

## Abstract

In this study, the titanium–gadolinium quantum dots (TGQDs) were novel, first of its type to be synthesized, and fully characterized to date. Multiple physical characterization includes scanning electron microscopy (SEM), scanning electrochemical microscope (SCEM), x-ray fluorescence, spectrophotometry, and dynamic light scattering were carried out. The obtained results confirmed appropriate size and shape distributions in addition to processing optical features with high quantum yield. The synthesized TGQD was used as a fluorescent dye for bacterial detection and imaging by fluorescent microscopy and spectrophotometry, where TGQD stained only bacterial cells, but not human cells. The significant antibacterial activities of the TGQDs were found against a highly pathogenic bacterium (*Staphylococcus aureus*) and its antibiotic resistant strains (vancomycin and methicillin resistant *Staphylococcus aureus*) using growth curve analysis and determination of minimum inhibitory concentration (MIC) analysis. Live/dead cell imaging assay using phase-contrast microscope was performed for further confirmation of the antibacterial activity. Cell wall disruption and release of cell content was observed to be the prime mode of action with the reduction of cellular oxygen demand (OD).

## 1. Introduction

Antibiotic resistance in bacteria is a major challenge to medical sciences and the priority list by World Health Organization (WHO) categorizes it into critical, high, and medium [1]. Research for the past two decades have produced only two new types of antibiotics: lipopeptides and oxazolidinones, although majorly overcoming antibiotic resistance has not been achieved yet. The present situation of bacterial infections is considered to be a threat to human society due to the evolving strains of *Staphylococcus aureus* with resistance to broad-range antibiotics such as methicillin and vancomycin [2]. The infections caused by the resistant bacteria are usually treated with existing antibiotics, which usually exerts side effects with their incomplete dosage and improper use. The overuse and misuse of antibiotics are the key factors contributing to antibiotic resistance [3]. The overuse of antibiotics, especially taking antibiotics even when they are not the appropriate treatment, promotes antibiotic resistance [4]. On the other hand side, the effects can be varied, for example, vomiting, nausea (feeling like one may vomit), diarrhea, bloating and indigestion, abdominal pain, loss of appetite, a raised, itchy skin rash (urticaria, or hives), coughing, wheezing, tightness of the throat, feeling lightheaded or faint, breathing difficulties such as fast, shallow breathing, wheezing, a fast heartbeat, clammy skin, confusion and anxiety, collapsing or losing consciousness [5]. Furthermore, some antibacterial agents can elicit adverse effects against the hepatic system, where the types of liver damage induced by antibacterial agents cover cytotoxic, injury, cholestatic injury, mixed cytotoxic and cholestatic injury, steatosis, chronic, active hepatitis, and cirrhosis [6]. This further increases the complexity in the development of the resistance against these molecules, assisting it in being a global health problem [7,8]. Thus, the importance of developing new treatment strategies or alternatives for antibiotics against these multidrug resistant bacterial strains are the major demands in 21st century medical science [9,10,11]. To overcome the antibiotic resistance and absolute necessity of finding an alternative to existing antibiotics with negligible toxicity, proper biocompatibility, and fulfil the needs of clinical research, novel antibacterial agents like nanoparticles, peptides, metabolites, oligonucleotides, and other biogenic or chemical compounds have been introduced [8,12,13,14,15,16,17]. Nanoparticles, nowadays, are the focus of medical sciences with various applications like drug delivery, tracking, and alternatives to antibiotics such as nanomedicine [18,19,20]. However, nanoparticles also have adverse effects toward human health. Nanoparticles exert various kinds of toxicity, they can be cancerous, affect our immune system, liver, spleen, can generate cellular ROS, damage our DNA, or they can also affect our metabolism through blocking or disrupting various enzymatic pathways [21,22]. 

The detection and imaging of bacteria by staining has been a reliable method for more than a century. Commercial unavailability of any bacteria specific fluorescent stain has limited the process of fluorescent labelling. Most of the existing stains follow a general mode of action, where it stains almost all types of cells including mammalian cells, or it is artificially designed for specific target staining. For example, BacLight green is a bacterial specific stain that contains SYTO9, frequently used in live/dead cell imaging of bacteria, but SYTO9 is an intercalating membrane permeable green stain that stains all cells containing nucleic acid [23]. Moreover the vital stain, fluorescein diacetate (FDA) is based on intracellular hydrolysis of FDA and in the case of living cells, the non-fluorescent FDA is converted into fluorescein, a green fluorescent compound, indicating the cell viability [24,25]. The detection of bacteria in human tissue during infection development, or the host pathogen interaction study by using a fluorescent dye is quite challenging due to the generalized mode of action of commercialized organic dyes. Thus, the necessity of developing a bacteria specific stain to improve the bacterial detection and imaging is required and long awaited. 

Nanoparticles (NPs) can be derived from various sources in a desirable nanometer (nm) size. Due to their enhanced retention and permeability, NPs have a broad range of clinical applications along with their ability to accumulate the sites of infection by enhancing retention and the permeability effect [26]. NPs with few modifications help in site specific drug delivery by reducing target toxicity [27]. Quantum dots (QDs) are a type of nanoparticles with the ability to fluoresce, which is helping in the development of new detection and imaging techniques [27,28,29]. A few novel QDs have been reported to have antibacterial [30,31,32] and anticancer properties [33,34], which elevates their value even more in the field of biomedicine with some drawbacks like toxicity, inflammation, and stability [27]. Titanium dioxide (TiO_2_) based nanocomposites have been reported to have antimicrobial activity against *Escherichia coli*, *Staphylococcus aureus*, *Streptococcus sobrinus*, *Pseudomonas putida*, *Pseudomonas aeruginosa*, and *Listeria innoculim* due to the production of reactive oxygen species (ROS), which can inactivate organic and inorganic pollutants and consequently inactivate the microorganisms, even where environmental and economic factors have raised their volume [35,36,37,38]. The gadolinium based nanoparticles also have antimicrobial activities against *Bacillus subtilis*, *E. coli*, and *Staphylococcus aureus* [39,40]. Though both gadolinium and TiO_2_ nanoparticles are biologically active, no study has tested their real implications and effects against vancomycin resistant *Staphylococcus aureus* (VRSA) and methicillin resistant *Staphylococcus aureus* (MRSA) yet.

In this study, we used titanium and gadolinium together to synthesize a titanium–gadolinium quantum dot (TGQG), the first of its kind, and its characterization was undertaken using dynamic light scattering, spectrophotometry, X-ray fluorescence, scanning electrochemical microscope (SECM), and scanning electron microscope (SEM). Thereafter, TGQD was used as a stain in the detection of VRSA using a fluorescence microscope. Furthermore, the antibacterial activity was tested against the pathogenic strains of vancomycin resistant *S. aureus* (VRSA), methicillin resistant *S. aureus* (MRSA), and the mechanism of action was also investigated. Finally, the cytotoxicity test was performed and conclusions were drawn.

## 2. Materials and Methods

### 2.1. Chemicals

2-Pyridinecarboxaldehyde, diethylene triamine, gadolinium (III) nitrate, titanium (IV) butoxide, isopropanol, and methanol were obtained from Sigma-Aldrich (St. Louis, MO, USA). Muller Hinton broth (MH) was obtained from HiMedia Laboratories Pvt. Ltd., Thane, India.

### 2.2. Chemicals and Synthesis of Titanium–Gadolinium Quantum Dots (TGQDs)

The preparation of the gadolinium Schiff base was according to our previous studies with necessary modification [28]. Initially, 1.08 mL of diethylene triamine and 1.9 mL of 2-pyridinecarboxaldehyde were mixed and heated under reflux in 35 mL of methanol (MeOH) for 6 h. To prepare the desired solution of Schiff base [(2-[(E)-2-pyridylmethyleneamino]-N-[2-[(E)-2-pyridylmethylene-amino]ethyl]-ethanamine)], the solution was cooled and the volume was made up to 50 mL using MeOH. In a separate beaker, 5 mL of gadolinium nitrate aqueous solution (90 mg/mL) was mixed with 10 mL of MeOH, which was subsequently mixed with 5 mL solution of the Schiff base. Finally, the solutions were mixed on hot plate magnetic stirrer for 2 h at 40 °C and the volume was made up to 100 mL with deionized water. The Gadolinium (Gd)–Schiff base solution was stored at 25 °C. To prepare the titanium dioxide (TiO_2_) solution, 50 mL of isopropanol and 50 µL of titanium butoxide was put into a glass beaker and placed on a magnetic stirrer for 48 h, and the clear solution turned into a milky white solution without precipitation. 

Then, the aqueous phase synthesis of quantum dots was carried out by mixing the Gd–Schiff base solution with TiO_2_ in a beaker. The Gd–Schiff base and TiO_2_ solutions were mixed in the ratio of 1:1. Later, 2 mL of the solution was taken in a glass tube and heated for 10 min at 80 °C, 300 W, (ramping time, 10 min) under microwave irradiation (Multiwave 3000, Anton Paar GmbH, Graz, Austria) to prepare the TG quantum dots. The samples were filtered using 3 kDa (Amicon Ultra 0.5 mL centrifugal filters) filters and further filtered through a 0.22 μm membrane. To remove the unreacted initiators, the solution was dialyzed against deionized water several times, then the TGQD were dried under a vacuum dry system. 

### 2.3. Characterization of TGQD

The prepared TGQDs were visualized under a UV transilluminator at excitation wavelengths (*λ*_ex_) of 312 nm and 270 nm (Transilluminator Multiband TFX-35.MC, Torcy, France). The fluorescence and absorbance spectra of the TG QDs were obtained using a microplate (UV plate, 96 well; Corning Incorporate, Corning, NY, USA) in Tecan Infinite m200 PRO (Männedorf, Switzerland). A total of 100 μL of the samples were used for the measurements. The fluorescence spectrum was measured using an excitation wavelength (*λ*_ex_) of 230 nm, the emission wavelength (*λ*_em_) range was 280–850 nm, and the absorbance spectrum of the TGQDs was measured from 230 to 1000 nm. The QDs were observed under a fluorescence microscope with a UV filter for visualization. The photoluminescence quantum yield of the TGQDs was determined using the reference as rhodamine 6G according to a reported protocol [41].

The scanning electron microscopy (SEM) instrument (TESCAN Company, Brno, Czech Republic, EU) was used to observe the TGQDs under the following conditions: high vacuum mode (10–3 Pa), voltage of 15 kV, and work distance of 3 mm [28].The scanning electrochemical microscopy (SECM) measurements were performed using a CHI 900 setup (CH Instrument Inc., Austin, TX, USA). A Pt ultramicroelectrode (*d* = 10 mm) was the SECM tip (RG factor = 10), a bare glassy carbon (GC) covered with 10 µL Nafion 1%, and a GC immobilized with TGQDs and covered with 10 µL Nafion 1% served as the SECM substrates. The tip was positioned near the substrate using the probe-approach curve (PAC) technique and a potential of 500 mV was applied to the tip with a scan rate of 0.5 µm/s. A platinum wire was the counter electrode and the reference electrode was Ag/AgCl (3 M KCl). A solution of 1 mM of ferrocenemethanol (FcOH) in 0.1 M KCl was used as the redox mediator. All potentials were referred to the reference electrode.

TGQDs were also characterized using an elemental analyzer SPECTRO XEPOS energy dispersive x-ray fluorescence (ED-XRF) spectrometer (SPECTRO Analytical Instruments GmbH, Kleve, Germany) equipped with a 10 mm^2^ Si-Drift Detector with Peltier cooling and a 75 µm Be side window was employed. The instrument uses a Pd-target end window tube at a maximum power of 50 W and a maximum voltage of 50 kV. Spectral resolution of the instrument (FWHM) was <170 eV for Mo Kα (measured under input count rate 10,000 pulses). SPECTRO XEPOS was operated and data were evaluated by means of the software Spectro X-Lab Pro, Version 2.5, Kleve, Germany. For the excitation of light elements (Mg–V, 25 kV), a HOPG (highly oriented pyrolithic graphite) crystal target was used. For the determination of heavier elements, a Mo secondary target (Cr–Y, Hf–U, 45 kV) and Al_2_O_3_ polarization target (Zr–Ce, 49.5 kV) were used. The sample (1.0 mL) was dried directly at 70 °C in a sample cup (32 mm in diameter) on polypropylene thin-film (Specac Ltd., Kent, UK) and measured in vacuum using the so-called Turboquant method (fundamental parameters method) [42]. Fourier transform infrared (FTIR) spectra were measured using a Thermo Scientific Nicolet iS5 spectrometer (Thermo Fisher Scientific, Waltham, MA, USA) equipped with an iD5 Diamond ATR accessory over a wave number range of 4000–550 cm^–1^. The average size of the TG QDs, the size distribution, and zeta potential were determined by quasielastic laser dynamic light scattering (DLS) with a Malvern Zetasizer (NANO-ZS, Malvern Instruments Ltd., Worcestershire, UK). Initially, 1.5 mL of an aqueous solution of TGQDs (1 mg/mL) was poured into a polystyrene latex cell and measured at a temperature of 25 °C with a detector angle of 173°, a wavelength of 633 nm, a refractive index of 0.30, and a real refractive index of 1.59 [28].

### 2.4. Application of TGQD on Bacteria for Detection and Killing

The bacterial strains *Staphylococcus aureus (S. aureus)* (NCTC 8511) [43,44], vancomycin resistant *Staphylococcus aureus* (CCM 1767) [19,45], and methicillin resistant *Staphylococcus aureus* (MRSA) ST239:SCCmec IIIA) [46] were obtained from the Czech Collection of Microorganisms, Faculty of Science, Masaryk University, Brno, Czech Republic and from England, with the cooperation of the University of Cambridge [19,43,44,45,46]. The bacterial cells were cultivated in Muller Hinton (MH) broth medium, pH 7.4. The bacterial cultures were cultivated overnight at 37 °C in a shaking incubator. Bacterial optical density was adjusted to 0.1 absorbance (0.5 MacFarland standards) at 600 nm for the subsequent experiments [45,46,47,48,49,50].

The absorbance spectral scan was recorded of the bacterial sample for their detection by using Tecan Infinite m200 PRO immediately after preparing the samples. Bacterial culture was adjusted up to 0.012 absorbance (OD600) and incubated with TGQD for 5 min, then the bacterial sample incubated with TGQD was placed on a spectrophotometric plate and the plate was scanned under the Tecan Infinite m200 PRO. 

Furthermore, the bacterial detection via TGQD was carried out through microscopic analysis. The bacterial sample (VRSA) was stained with TGQD (15 µg/mL) and Images were obtained using an optical Olympus BX51 fluorescence microscope equipped with 40× phase contrast lens. 

Thereafter, further confirmation was obtained by treating bacteria, bacteria–human cell co-culture with TGQD, and microscopic images were captured. For the co-culture and the bacterial sample, VRSA was added in a 24-well cell culture plate in the presence of PNT1A (human cells) cells with 60% confluency, incubated for 1 h at 37 °C. The co-culture system containing bacteria and human cells together was stained by TGQD (15 µg/mL), then the plate was used for microscopy. The microscopy was performed by using an microscopic inverted Olympus IX 71S8F3 fluorescence microscope (Olympus Corporation, Tokyo Japan), which was equipped with an Olympus UIS2 series objective LUCplanFLN 40X (N.A. 0.6, WD 2.7–4 mm, F.N.22), and a mercury arc lamp X-cite 12 (120W; Lumen Dynamics, Mississauga, ON, Canada) was used. As the control, bacterial cells and the human cell line PNT1A were also stained with TGQD and observed under a microscope.

The bactericidal effect of TGQD was analyzed by scanning electrochemical microscopy (SECM). where a Petri dish containing the VRSA treated with TGQD was subjected to the SECM study. The Petri dish was coated with Poly-L-Lysine and then the VRSA cells were added to the Petri dish for their attachment with the Poly-L-Lysine coated Petri dish surface. The Petri dish was filled with MH Broth, which was composed of beef infusion form 300.00 g/L, casein acid hydrolysate 17.50 g/L, starch 1.50 g/L, and KCl 7.45 g/L. The probe electrode (Pt 10 µm) was inserted in the Petri dish and the cyclic voltammetry (CV) was recorded. 

Standard isolates of *S. aureus*, VRSA, and MRSA were cultivated and used for this experiment. All bacterial isolates were grown in Mueller Hinton broth in a shaking incubator at 37 °C. The susceptibility of bacterial cultures against TGQD detection was performed by the unaided eye [45,46,51,52]. The minimum inhibitory concentration (MIC) is the lowest concentration that inhibits bacterial growth. The MIC of TGQD was obtained by adding TGQD from lower to higher concentrations in microplate wells, mixed with bacterial cultures (0.1 O.D. equivalent to 0.5 MacFarland), and incubated at 37 °C for 24 h. The final working concentration gradient of TGQD in the microplate wells were 45 µg/mL, 55 µg/mL, 62.5 µg/mL, 85 µg/mL, and 125 µg/mL. The lowest concentration of TGQD added in the microplate well and the microplate well showed almost no bacterial growth (transparent medium with no turbidity), which was counted as the MIC value of TGQD against that specific bacterium. The control was the bacteria without TGQD treatment.

The growth curve analysis for *S. aureus*, VRSA, and MRSA were performed for further confirmation of antibacterial activity of TGQD. Standard isolates of *S. aureus*, VRSA, and MRSA were cultivated and used for this experiment. All bacterial isolates were grown in MH broth in a shaking incubator at 37 °C. The control was the bacterial culture with no TGQD treatment. The antibacterial activity of the TGQDs were measured by Bioscreen C MBR (Oy Growth Curves Ab Ltd., Helsinki, Finland) for 24 h at 37 °C using a multichannel pipette system in the 10 × 10 Honeycomb optical microplate well systems (Oy Growth Curves Ab Ltd., Helsinki, Finland) [51,53,54,55,56]. Different concentrations of TGQDs (45 µg/mL, 55 µg/mL, 62.5 µg/mL, 85 µg/mL, 125 µg/mL) were added to bacterial cultures (0.1 absorbance equivalent to 0.5 McFarland) in the microplate wells. The microplate with the samples was then incubated in the growth curve analyzer (Bioscreen C MBR) for 24 h and the results were evaluated accordingly [46]. 

Furthermore, the cell leakage assay was carried out. The bacterial sample was treated with TGQD and incubated at 37 °C for 4 h, which showed the cells treated with the TGQD were precipitated after 4 h. The treated samples as well as the untreated samples were centrifuged for cell debris removal and the supernatant was separately kept in a micro-centrifuge tube for quantification of DNA and RNA by a spectrophotometer (Tecan Infinite m200 PRO). The wavelength of 260 nm was used for the DNA and RNA quantification. For the DNA quantification, the samples were treated with RNase, and for the RNA quantification samples, we treated them with DNase, and purified them in a column purification system. After column purification, the absorbance spectra were recorded at 260 nm. The presence of DNA and RNA were calculated according to a 260/280 ratio. According to the 260/280 ratio, ~1.8 is generally accepted as pure for DNA and ~2.0 is generally accepted as pure for RNA [57,58,59,60,61]. The DNA in the supernatant was used as a template with 16S rRNA primers (16S Forward-ACTGGGATAACTTCGGGAAAC, and 16S reverse-CAGCGCGGATCCATCTATAA) to perform PCR amplification, and the confirmation was done using agarose gel electrophoresis with 1.3% agarose gel [45]. The positive control was genomic DNA and the negative control was the supernatant from the untreated bacterial sample.

To confirm the presence of all types of live cells (bacterial and human cells), SYTO9 was used. Thereafter, an optical Olympus BX51 fluorescence microscope equipped with a 40× phase contrast lens was used to study the antibacterial activity of the TGQD against VRSA. Initially, the samples were incubated with bacteria and TGQDs (respective MIC) for 4 h at 37 °C in a shaking incubator. Two different kinds of fluorescent dyes were used: SYTO9 and propidium iodide (PI) for live and dead cell staining in equal proportion. Fluorescence dyes were added to the sample and observed under microscope. 

### 2.5. In Vitro Cytotoxicity Testing Assay

The MDA-MB-231 (mammary gland adenocarcinoma cells) and HBL-100 (mammary gland epithelial cells) human cell line were used to study the cytotoxicity of TGQDs. The MTT 3-(4,5-dimethylthiazol-2-yl)-2,5-diphenyltetrazolium bromide) assay was used to study the cell viability. The cells were maintained in RPMI-1640 medium with 10% fetal bovine serum, supplemented with penicillin (100 u/mL) and streptomycin (0.1 mg/mL). In a microtiter plate, each well was filled with 5000 cells in 50 µL medium, followed by 24 h incubation at 37 °C with 5% CO_2_. After 24 h incubation with TGQDs, 10 µL of MTT (5 mg/mL in PBS) was added, and incubated for 4 h at 37 °C with 5% CO_2_. When this incubation period was over, MTT containing medium was replaced and 100 µL of 99.9% dimethyl sulfoxide (v/v) was added for 5 min. The absorbance was taken at 570 nm by an Infinite m200 PRO reader [46,62] 

## 3. Results and Discussion

### 3.1. Synthesis and Characterization of TGQDs

**Figure 1:**
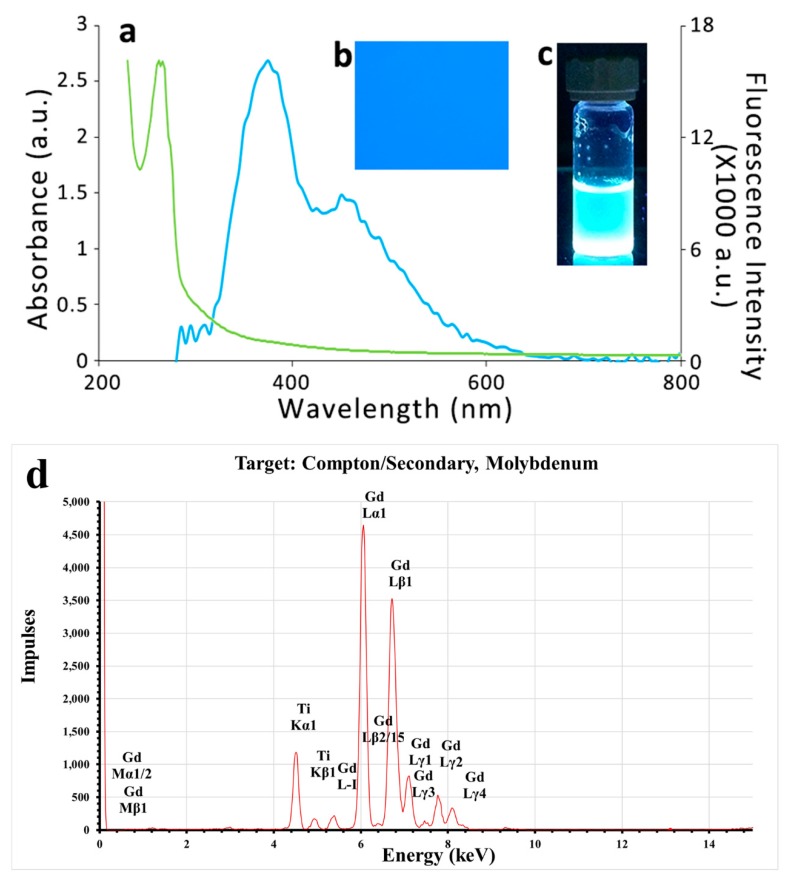
(**a**) The fluorescence spectrum is given in the blue line and the absorbance spectrum is given in the green line. (**b**) Titanium-Gadolinium Quantum dot (TGQD) observed under the UV filter of the fluorescence microscope. (**c**) TGQD shows a bluish white color under UV transilluminator; (**d**) X–ray fluorescence (XRF) spectrum confirms that the synthesized TGQDs contained both gadolinium and titanium metal.

**Figure 2:**
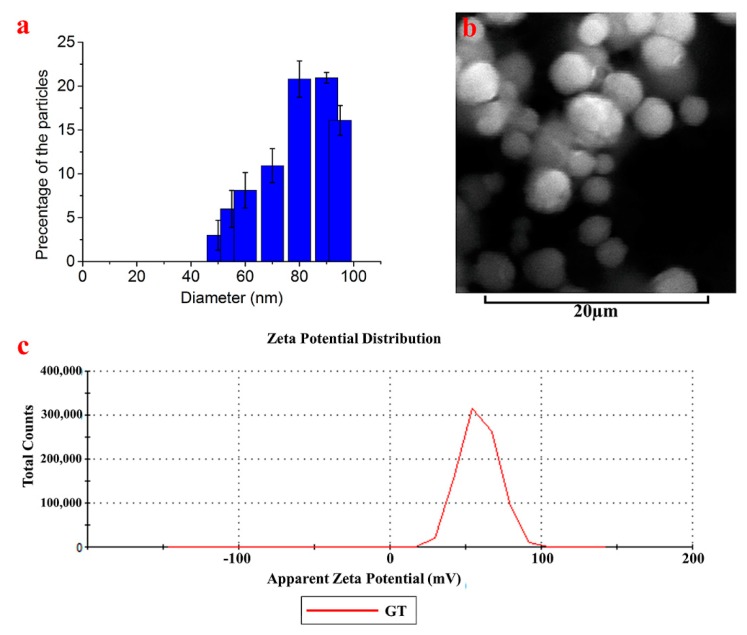
(**a**) Zeta size of the TGQD. (**b**) SEM analysis of TGQD with 20 µm scale bar;.(**c**) Zeta potential of TGQDs. Data represent the mean ± SD, *n* = 5.

The characterization was initiated by measuring the fluorescence and absorbance spectra of the TGQDs, as shown in Figure 1a. The Gd–Schiff base was mixed with TiO_2_ in a 1:1 ratio and cooked at 80 °C to form TGQDs, which when observed under microscopy showed a blue field due to its high fluorescence as observed under a UV filter (Figure 1b). The TGQD under UV transilluminator (*λ* = 312 nm) showed high fluorescence intensity with a bluish white color (Figure 1c). The fluorescence emission maximum and the absorbance maximum of the TGQDs were observed at *λ* = 375 nm and *λ* = 262 nm, respectively. Furthermore, it was characterized by x-ray fluorescence spectrophotometry using the secondary target as Mo. The results indicate specific peaks that showed the presence of Gd and Ti in the sample, as shown in Figure 1d. Moreover, the particle size distribution, average particle size, and the zeta potential of the prepared TGQDs were analyzed using SEM and DLS (Figure 2). The results revealed that the size of the TGQDs were found to be in the range of 45 ± 2 nm to 95 ± 2 nm (Figure 2a) and the value of the zeta potential showed that the value was highest at 58.7 ± 0.13 mV (Figure 2c). The size determined by DLS was in good agreement with the SEM image and data, as shown in Figure 2b.

**Figure 3:**
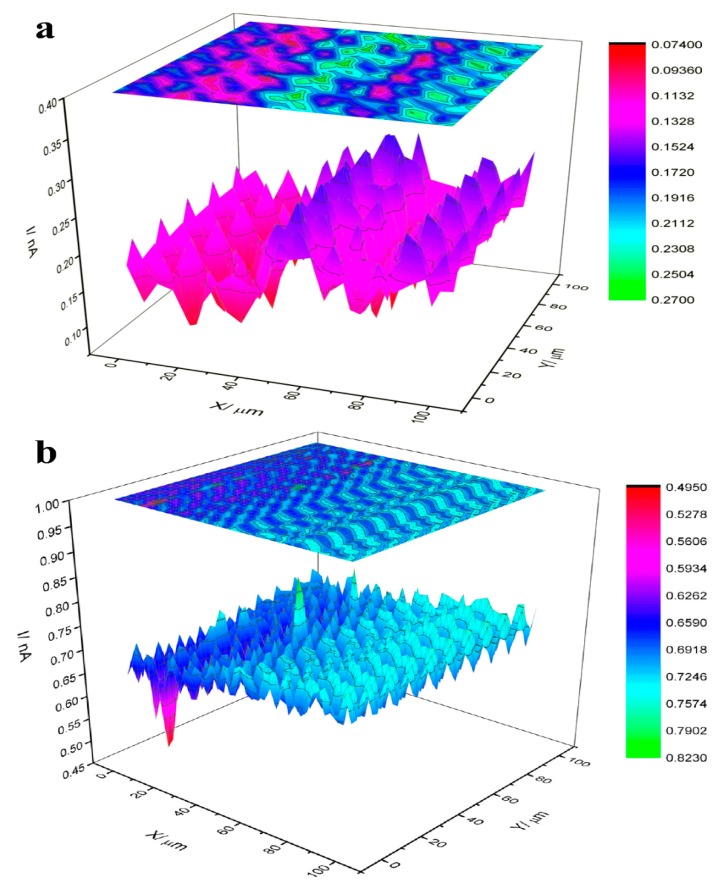
(**a**) Scanning electrochemical microscopy (SECM) image of glassy carbon electrode covered with Nafion. (**b**) SECM image of glassy carbon immobilized with QDs and covered with Nafion. The applied parameters: the tip electrode scan rate: 20 µm/s, the applied potential on tip: 0.5 V, the tip—substrate distance: 10 µm, quiet time: 30 s, the scanned surface area 100 µm × 100 µm. The measurements were carried out in a solution of 1 mm FcOH and 0.1 M KCl.

Finally, the FTIR spectrum of TGQD (Figure S3) indicates the presence of the different vibrations of the functional group, showing the presence of all the bond and functional groups present in the gadolinium nitrate and titanium butoxide, initial component of TGQD (Figures S1 and S2). In titanium, the functional group NO_3_ has asymmetric and symmetric stretching from 1369 to 1381 cm^−1^ and 1340 to 1267 cm^−1^ [63]. In the case of titanium butoxide absorptions at 1125 cm^−1^ (Ti–O–C vibration), the intensity of the bands was from 1490 to 1340 cm^−1^ [64]. The SECM images obtained are shown in Figure 3. The scale of the current measured on the bare glassy carbon (GC) was from 0.09 to 0.2 nA. The GC immobilized with TGQD showed a current range mainly from 0.6 to 0.7 nA. Thus, it can be concluded that the current interval in both cases was the same (almost 1 nA). Hence, the inhomogeneity of both electrodes is comparable. It can be concluded that the homogeneity of the both electrodes is mainly affected by the Nafion layer. However, the current increased significantly in the presence of the TGQD. This can be attributed to the good electrochemical conductivity of the synthesized TGQD, which is due to low electrochemical resistance of their metallic components. Moreover, the charge of the QDs may influence their electrochemical conductivity. However, we found that the TGQDs were water soluble and showed high stability (fluorescence measurement) in solution, even after four months from its prepared time.

### 3.2. Detection of Bacteria

Furthermore, according to the spectrophotometry it is clear that the bacterial cells were detected by applying TGQDs, where bacterial cells presented a 0.012 O.D. value, whereas absorbance from only bacteria and only QDs were lower than the QD mixed sample. Lower concentrations (0.012 O.D. concentration) of the bacterial sample were detected through the spectrophotometric method (Figure S4). The bacterial sample was stained with TGQD, which gave a high bluish white fluorescence under the UV filter of the fluorescence microscope (Figure S5).

**Figure 4:**
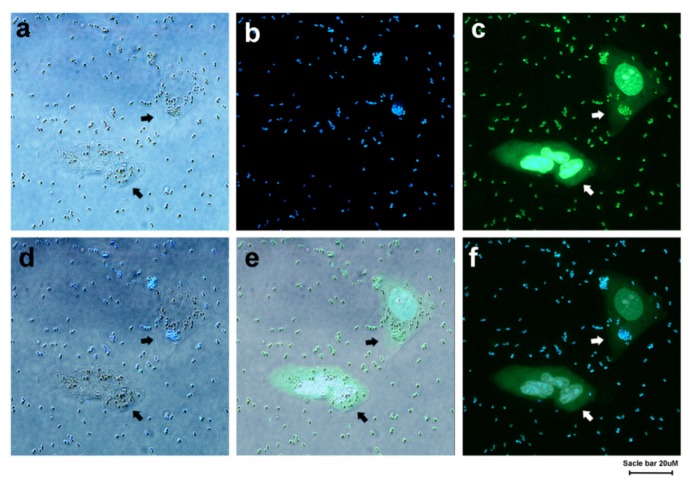
The microscopic images. (**a**) VRSA in presence of PNT1A cells; (**b**) VRSA in presence of PNT1A cells stained with TGQDs and imaged under UV filter; (**c**) VRSA mixed with PNT1A cells stained with SYTO9 and imaged under fluorescence microscope through 485/498 filter; (**d**) Merged image of (a) and (b); (**e**) Merged image of (a) and (c); (**f**) Merged image of (b) and (c).

Detection (through imaging) of the VRSA in the presence of PNT1A (coculture imaging) was carried out under fluorescence microscopy. The samples were stained with a very small amount (10 to 15 μg/mL) of TGQD. The VRSA cells in the presence of PNT-1A were stained with TGQD and SYTO9 to understand the dye specificity (whether TGQD is specific toward bacteria or not) toward bacteria and human cells. The bright field image (Figure 4a) showed the presence of bacteria and human cells (indicated by arrow) in the field of observation. Figure 4b shows that TGQD positively stained the bacterial cells, but PNT1A cells were unstained and not visible in the field. In Figure 4c,e (merged image of Figure 4a,c), it can be seen that the SYTO9 stained both the VRSA and the PNT1A cells, which confirmed that SYTO9 does not have a cell specific staining nature. In contrast, TGQD specifically stained the bacterial cells, which was clearly visible in Figure 4b,d (merged image of Figure 4a,b) and f (merged image of Figure 4b,c). Finally, from Figure 4f (the merged image of Figure 4b,c), it can be clearly concluded that STYO9 is neither specific toward bacteria nor human cells (because both bacterial and human cells were stained), whereas the TGQD is a bacteria specific stain (only bacterial cells were stained). According to the best of our knowledge, there is no such TGQD that can stain only bacteria and not the human cells. This experimental data was further validated by control cell imaging (Figure S6) where TGQD stained VRSA cells showed high fluorescence under a fluorescence microscope, but the PNT1A cells were unstained and no fluorescence was observed. 

### 3.3. Antibacterial Activities

**Figure 5:**
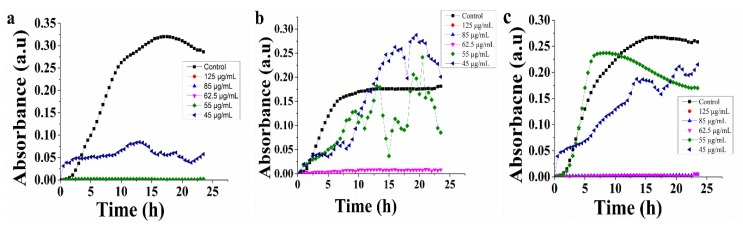
Analysis of growth curve for *S. aureus* (**a**)*,* VRSA (**b**), and MRSA (**c**) treated with TGQD. Data represent the mean ± SD, *n* = 5. VRSA = vancomycin resistant *S. aureus*.

The antibacterial activity of TGQDs was initiated by the determination of the MIC of TGQD, which was performed by the broth micro-dilution method against VRSA, MRSA, and *S. aureus*. The MIC of the TGQD against VRSA, MRSA, and *S. aureus* was obtained as 62.5, 62.5, and 55 µg/mL, as shown in Table 1. However, the TGQD concentrations below the respective MIC value showed turbidity in the solutions, proving the presence of bacteria in the solutions. Thereafter, different concentrations of the TGQDs (125 µg/mL, 85 µg/mL, 62.5 µg/mL, 55 µg/mL, and 45 µg/mL) were used to further understand the growth curves of respective bacteria in the presence of TGQD [45,51]. The 24 h growth curve of *S. aureus* showed more than 97% inhibition up to 55 µg/mL, but concentrations below showed lower inhibitory effects, as shown in Figure 5a. However, in the case of VRSA and MRSA, the inhibition was significant up to more than 98% at 62.5 µg/mL, but concentrations below showed no inhibitory effects, as shown in Figure 5b,c. Depending on the previous research, vancomycin resistant strains (contains VanA clustered gene) growth was inhibited with the use of 512 µg/mL of vancomycin [65,66], but this is not sufficient for preventing or curing infections by vancomycin resistant strains. Thus, it can be concluded that our synthesized TGQDs are novel and biologically active antibacterial compounds that can inhibit the growth of resistant strains of *S. aureus* almost completely by using 62.5 µg/mL concentrations. Thereafter, the mechanism of action of TGQD was studied. 

### 3.4. Mechanism of Actions

**Figure 6:**
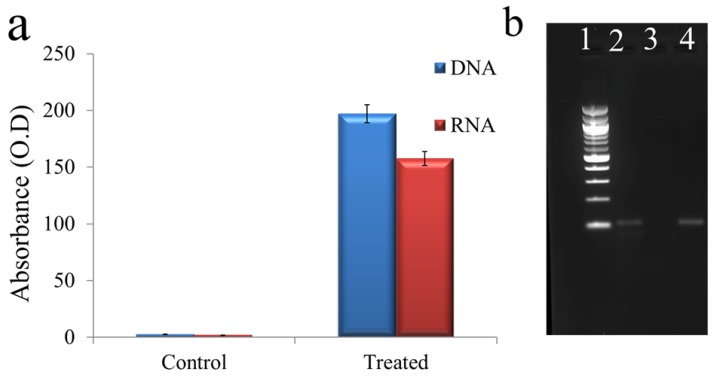
(**a**) DNA and RNA concentration measurement from the untreated sample and TGQD treated sample. (**b**) Cell wall damage and leakage assay where (1) 100 bp ladder, (2) PCR product of 16S rRNA of supernatant of VRSA cells treated with QDs, (3) PCR product of 16S rRNA of supernatant of VRSA cells not treated with TGQDs, and (4) PCR product of 16S rRNA of genomic DNA of VRSA cells treated with TGQDs.

Bacterial cells are negatively charged and our synthesized QDs were highly positively charged, for this reason, TGQD had a high binding affinity with the bacterial cells. Therefore, to study and understand the mechanism of action, VRSA cells were incubated with 62.5 µg/mL of TGQD for 4 h at 37 °C in a shaking incubator and the cell leakage assay was performed. The supernatant was used to detect the presence of DNA and RNA after treatment with TGQD. The presence of DNA and RNA was detected by spectrophotometry, as shown in Figure 6a, and further confirmed by the presence of bands around 109 bps in the agarose gel after PCR amplification of the 16S rRNA sequence using the supernatant as the DNA template (Figure 6b). The positive control showed no band, whereas the negative control showed the presence of a band in the same place as seen in the case of the treated samples. Thus, the result showed that the treatment of VRSA with TGQD caused leakage of the cells, which may by interacting with the cell membrane, causing the release of inner components containing nucleic acid into the supernatant. As a result, the PCR amplification showed amplified DNA product.

**Figure 7:**
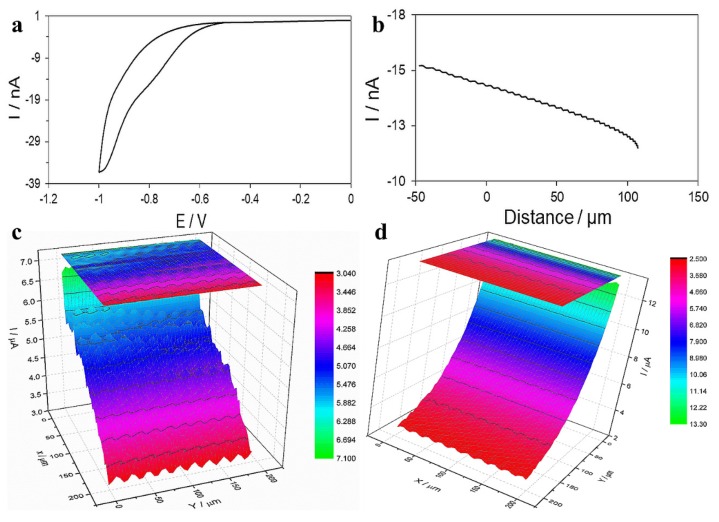
(**a**) dissolved oxygen reduction; (**b**) drop down percentage of probe; (**c**) represents high oxygen consumption by the bacteria and a decrease in the magnitude; (**d**) increased magnitude due to the absence (death) of bacterial population.

As shown in Figure 7a, a reduction peak could be observed around −0.8 V, which was attributed to the reduction in dissolved oxygen. This reduction peak is suitable to track the biological species that consume oxygen [67]. Thus, the potential was set at −0.8 V and the probe approach curve was recorded on a part that was removed from the bacteria. Since the Petri dish is an insulating material, a decrease in the current magnitude was expected when moving the probe toward the Petri dish. When the current dropped to 75% of its initial magnitude, the probe was stopped (Figure 7b). Theoretically, at this current magnitude, the distance between the probe to the bottom of the Petri dish is around 10 µm [68]. When the probe was positioned at an appropriate distance to the Petri dish, it was moved to part of the Petri dish with bacteria. Then, the surface scanning (200 µm × 200 µm) was carried out. As seen in Figure 7c, the current magnitude was mainly between the range of 3.5 to 6.0 nA. Furthermore, the surface seems to be rough, which is due to the presence of the bacteria. It must be noted that due to the oxygen consumption, the current magnitude decreased when the probe was placed above the bacteria, hence the roughness of the surface was due to the presence of bacteria and its rough texture. The effect of the TGQDs on the bacteria was also studied by the addition of TGQDs (50 µg/mL) to the same Petri dish containing the bacteria. After 5 min, when the interaction of the TGQDs and the bacteria was ensured, the same surface of the Petri dish was scanned. As can be observed in Figure 7d, the current magnitude ranged from 3.5 nA to 11.0 nA and with scanning, the current gradually increased. The increase in the current magnitude was attributed to the increase in dissolved oxygen concentration. It can be then assumed that the interaction of the TGQDs with the bacteria resulted in the death of bacteria. Then, the bacteria no longer consume dissolved oxygen. Moreover, the surface showed less roughness when compared to that in Figure 7c. When the bacteria die, they detach from the surface of the Petri dish and float in the buffer. Thus, the surface of the Petri dish, which was less rough, was actually imaged after the addition of TGQDs.

**Figure 8:**
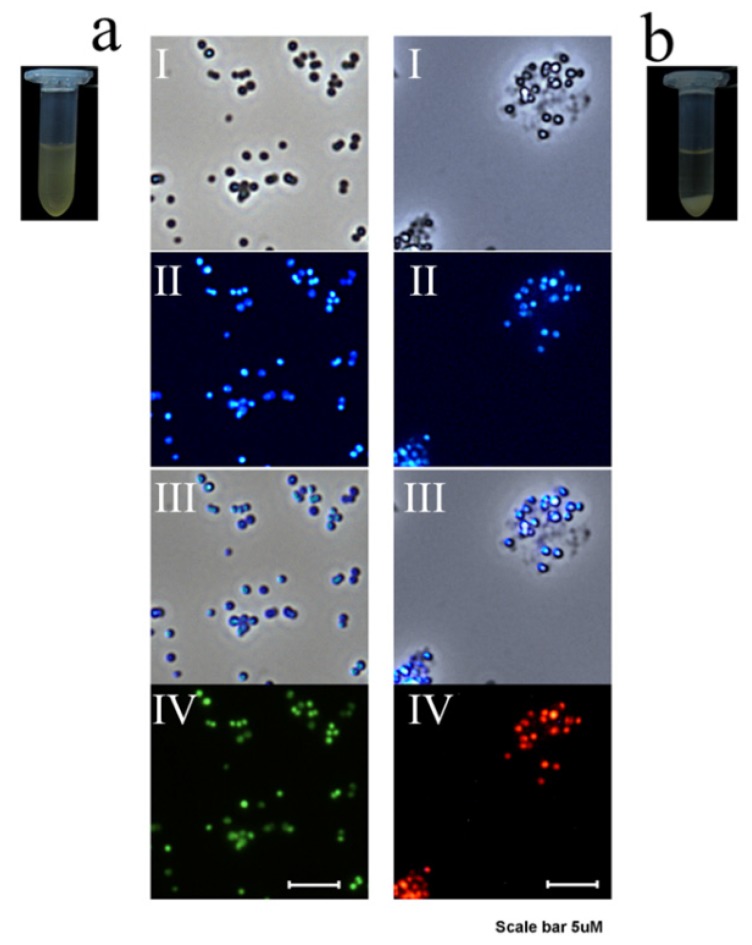
(**a**) TGQD treated VRSA at 0 h where (I) stands for the phase contrast image of VRSA, (II) stands for UV excitation image, (III) denotes ‘I’ and ‘II’ merged cells, and (IV) denotes SYTO9 (living cells green fluorescence) and propidium iodide (dead cells red fluorescence) stained cells. (**b**) TGQD treated VRSA in 4 h where (I) stands for the phase contrast image of VRSA, (II) stands for UV excitation image in phase contrast microscope, (III) denotes ‘I’ and ‘II’ merged cells, and (IV) SYTO9 and propidium iodide stained cells fluorescence image.

Finally, the VRSA was treated with TGQD to observe the viability of the cells by a live/dead cell assay using a fluorescence microscope equipped with phase contrast. The samples were stained with SYTO9 and propidium iodide (PI), which helps to differentiate the live and dead bacterial cells. The samples were treated with TGQD (125 µg/mL) and observed under the microscope after a time interval of 0 h and after 4 h of treatment. The images of the micro-centrifuge tubes had considerable change in the samples after 4 h of treatment, showing the precipitation of dead VRSA cells along with TGQDs at the bottom of the tube with a clear solution above. Furthermore, the microscopic images also agreed with the precipitated samples after 4 h of treatment. The phase contrast image for 0 h showed the presence of the VRSA in bright field, similarly, an identical image of the field under UV excitation proved the interaction of the VRSA with TGQD (both separately and in merged field), without any aggregations of bacteria, as seen in the micro-centrifuge tube. Moreover, the green fluorescence of the bacteria, which was due to the presence of SYTO9, proved the VRSA cells were in a viable state at 0 h. In the case of the 4 h treated sample, it showed aggregation of the bacterial cells in the phase contrast under bright field with the TGQD and under UV excitation also showed the bacteria in the identical position in that field. Further PI emitting red fluorescence confirmed that the cells were dead in that specific field, as shown in Figure 8**.** Thus, the TGQD not only detects both live and dead bacterial cells by staining both live and dead VRSA cells, but also showed considerable antibacterial effects against VRSA with time by interacting with the negatively charged cell wall of the bacteria. 

### 3.5. In Vitro Cytotoxicity Analysis

The cytotoxicity of TGQDs was estimated using the 3-(4,5-dimethylthiazol-2-yl)-2,5-diphenyltetrazolium bromide (MTT) assay with mammary gland epithelial cells (HBL-100) and mammary gland adenocarcinoma cells (MDA-MB-231) [69,70,71]. The viability was studied using different concentrations of TGQD, as shown in Figures S7 and S8. In the case of normal epithelial cells (HBL-100), the TGQDs did not show any sign of toxicity at any concentration tested. Furthermore, the adenocarcinoma cells showed a slight reduction in viability, which was almost negligible and thus no prominent cytotoxicity was seen due to the TGQDs. Thus, the results showing the high viability of normal cells, but slight reduction in viability of cancer epithelial cells in the presence of TGQD at a higher concentration, proved that they are biocompatible and non-toxic toward normal human cells in vitro with some effects against cancer cells.

## 4. Conclusions

In the present study, the synthesis of the TGQDs was itself a novelty, as it was the first of its kind to be synthesized to date. The characterizations of the TGQD helped to obtain the biophysical and biochemical information, stating a size below 100 nm, the charge of the TGQD was positive with the presence of both Ti and Gd in the QD, and the fluorescence and absorbance maxima of the TGQD was in the UV region with a high quantum yield of 29.3%. Subsequently, the TGQD was used to detect the VRSA in a co-culture of VRSA and PNT1A cells. The limitations of fluorescence dye (SYTO9) to stain specific bacteria were overcome by TGQD because there is no bacteria specific stain that can stain only bacteria and no other cells, but SYTO9 stains both bacteria and human cells. The TGQD stained the VRSA cells to emit bluish white fluorescence without staining the PNT1A cells, but SYTO9 stained both types of cells emitting green fluorescence. Furthermore, the TGQD inhibits by interacting with the bacterial cells causing cell leakage, and ultimately the release of the inner components. The antibacterial activities of the TGQD against *S. aureus* and its resistant strains showed strong inhibitory activities, which were validated by microscopic evidence. The mode of action was cell disruption and leakage of cytoplasmic content, which led to the precipitation and accumulation of bacterial cells with the amount of oxygen demand decreasing. However, it was also observed that the presence of any bacteria live or dead was detected by TGQD through fluorescence microscopic analysis. Finally, the cytotoxicity test of the TGQD showed no toxicity and proved the TGQD was biocompatible for normal human cells in vitro. Thus, the examined TGQD with biocompatibility and non-toxicity can be used as potential theranostics to detect and inhibit the presence of bacteria in different types of co-cultures or mixed samples with significant antibacterial activity. To the best of our knowledge, there is no such staining agent present that can stain only bacterial cells. In various research purposes like host pathogen interaction, study requires a specific staining agent. Thus, TGQD can be very useful for host pathogen interaction study. 

## Figures and Tables

**Table 1:** Determining the Minimum Inhibitory Concentration (MICs) of TGQD by the broth microdilution method.

Bacterial Strains	TGQD (µg/mL)
***S. aureus***	55
**MRSA**	62.5
**VRSA**	62.5

## Supplementary Materials

The following are available online at https://www.mdpi.com/2079-4991/10/4/778/s1, Figure S1: FTIR spectrum for gadolinium nitrate, Figure S2: FTIR spectrum for titanium butoxide, Figure S3: Synthesized TGQDs FTIR spectrum, Figure S4: Bacterial detection in low concentration, Figure S5: Bacterial cells stained with TGQD, Figure S6: Bacterial cells and Human PNT1A cells were stained with SYTO9 and TGQD,; Figure S7: MTT assay results for HBL-100, Figure S8: MTT assay results for MDA-MB-231.

## References

[1] Tacconelli E., Carrara E., Savoldi A., Harbarth S., Mendelson M., Monnet D.L., Pulcini C., Kahlmeter G., Kluytmans J., Carmeli Y. (2018). Discovery, research, and development of new antibiotics: The WHO priority list of antibiotic-resistant bacteria and tuberculosis. Lancet Infect. Dis..

[2] Foster T.J. (2017). Antibiotic resistance in Staphylococcus aureus. Current status and future prospects. FEMS Microbiol. Rev..

[3] Barker A.K., Brown K., Ahsan M., Sengupta S., Safdar N. (2017). Social determinants of antibiotic misuse: A qualitative study of community members in Haryana, India. BMC Public Health.

[4] Antibiotics: Are You Misusing Them?. https://www.mayoclinic.org/healthy-lifestyle/consumer-health/in-depth/antibiotics/art-20045720.

[5] NHS U. Side Effects of Antibiotics. https://www.nhs.uk/conditions/antibiotics/side-effects/.

[6] Westphal J.F., Vetter D., Brogard J.M. (1994). Hepatic side-effects of antibiotics. J. Antimicrob. Chemother..

[7] Dang C.N., Prasad Y.D.M., Boulton A.J.M., Jude E.B. (2003). Methicillin-resistant *Staphylococcus aureus* in the diabetic foot clinic: A worsening problem. Diabet. Med..

[8] Baptista P.V., McCusker M.P., Carvalho A., Ferreira D.A., Mohan N.M., Martins M., Fernandes A.R. (2018). Nano-Strategies to Fight Multidrug Resistant Bacteria-“A Battle of the Titans”. Front. Microbiol..

[9] Habiba K., Bracho-Rincon D.P., Gonzalez-Feliciano J.A., Villalobos-Santos J.C., Makarov V.I., Ortiz D., Avalos J.A., Gonzalez C.I., Weiner B.R., Morell G. (2015). Synergistic antibacterial activity of PEGylated silver–graphene quantum dots nanocomposites. Appl. Mater. Today.

[10] Alanis A.J. (2005). Resistance to Antibiotics: Are We in the Post-Antibiotic Era?. Arch. Med. Res..

[11] Sang Y., Blecha F. (2008). Antimicrobial peptides and bacteriocins: Alternatives to traditional antibiotics. Anim. Health Res. Rev..

[12] Edwards-Jones V., Rai M.K., Kon K.V. (2013). Chapter 1—Alternative Antimicrobial Approaches to Fighting Multidrug-Resistant Infections. Fighting Multidrug Resistance with Herbal Extracts, Essential Oils and Their Components.

[13] Kuete V., Rai M.K., Kon K.V. (2013). Chapter 3—Bioactivity of Plant Constituents against Vancomycin-Resistant Enterococci. Fighting Multidrug Resistance with Herbal Extracts, Essential Oils and Their Components.

[14] Ramírez Rueda R.Y., Rai M.K., Kon K.V. (2013). Chapter 2—Natural Plant Products Used against Methicillin-Resistant Staphylococcus aureus. Fighting Multidrug Resistance with Herbal Extracts, Essential Oils and Their Components.

[15] Barros C.H.N., Fulaz S., Stanisic D., Tasic L. (2018). Biogenic Nanosilver against Multidrug-Resistant Bacteria (MDRB). Antibiotics.

[16] Pal I., Bhattacharyya D., Kar R.K., Zarena D., Bhunia A., Atreya H.S. (2019). A Peptide-Nanoparticle System with Improved Efficacy against Multidrug Resistant Bacteria. Sci. Rep..

[17] Hemeg H.A. (2017). Nanomaterials for alternative antibacterial therapy. Int. J. Nanomed..

[18] Patra J.K., Das G., Fraceto L.F., Campos E.V.R., Rodriguez-Torres M.D.P., Acosta-Torres L.S., Diaz-Torres L.A., Grillo R., Swamy M.K., Sharma S. (2018). Nano based drug delivery systems: Recent developments and future prospects. J. Nanobiotechnol..

[19] Sur V.P., Kominkova M., Buchtova Z., Dolezelikova K., Zitka O., Moulick A. (2019). CdSe QD Biosynthesis in Yeast Using Tryptone-Enriched Media and Their Conjugation with a Peptide Hecate for Bacterial Detection and Killing. Nanomaterials.

[20] Thurn K.T., Brown E., Wu A., Vogt S., Lai B., Maser J., Paunesku T., Woloschak G.E. (2007). Nanoparticles for applications in cellular imaging. Nanoscale Res. Lett..

[21] Bahadar H., Maqbool F., Niaz K., Abdollahi M. (2016). Toxicity of Nanoparticles and an Overview of Current Experimental Models. Iran. Biomed. J..

[22] Crisponi G., Nurchi V.M., Lachowicz J.I., Peana M., Medici S., Zoroddu M.A., Grumezescu A.M. (2017). Chapter 18—Toxicity of Nanoparticles: Etiology and Mechanisms. Antimicrobial Nanoarchitectonics.

[23] Stocks S.M. (2004). Mechanism and use of the commercially available viability stain, BacLight. Cytom. Part A.

[24] Datta S., Sherman J.M., Bravard M.A., Valencia T., Gilman R.H., Evans C.A. (2014). Clinical Evaluation of Tuberculosis Viability Microscopy for Assessing Treatment Response. Clin. Infect. Dis..

[25] Kanade S., Nataraj G., Ubale M., Mehta P. (2016). Fluorescein diacetate vital staining for detecting viability of acid-fast bacilli in patients on antituberculosis treatment. Int. J. Mycobacteriol..

[26] Azzopardi E.A., Ferguson E.L., Thomas D.W. (2012). The enhanced permeability retention effect: A new paradigm for drug targeting in infection. J. Antimicrob. Chemother..

[27] Anderson D., Mj S. (2016). Nanotechnology: The Risks and Benefits for Medical Diagnosis and Treatment. J. Nanomed. Nanotechnol..

[28] Moulick A., Heger Z., Milosavljevic V., Richtera L., Barroso-Flores J., Merlos Rodrigo M.A., Buchtelova H., Podgajny R., Hynek D., Kopel P. (2018). Real-Time Visualization of Cell Membrane Damage Using Gadolinium–Schiff Base Complex-Doped Quantum Dots. ACS Appl. Mater. Interfaces.

[29] Ristic B.Z., Milenkovic M.M., Dakic I.R., Todorovic-Markovic B.M., Milosavljevic M.S., Budimir M.D., Paunovic V.G., Dramicanin M.D., Markovic Z.M., Trajkovic V.S. (2014). Photodynamic antibacterial effect of graphene quantum dots. Biomaterials.

[30] Li H., Huang J., Song Y., Zhang M., Wang H., Lu F., Huang H., Liu Y., Dai X., Gu Z. (2018). Degradable Carbon Dots with Broad-Spectrum Antibacterial Activity. ACS Appl. Mater. Interfaces.

[31] Garcia I.M., Leitune V.C.B., Visioli F., Samuel S.M.W., Collares F.M. (2018). Influence of zinc oxide quantum dots in the antibacterial activity and cytotoxicity of an experimental adhesive resin. J. Dent..

[32] Meikhail M.S., Abdelghany A.M., Awad W.M. (2018). Role of CdSe quantum dots in the structure and antibacterial activity of chitosan/poly ɛ-caprolactone thin films. Egypt. J. Basic Appl. Sci..

[33] Belletti D., Riva G., Luppi M., Tosi G., Forni F., Vandelli M.A., Ruozi B., Pederzoli F. (2017). Anticancer drug-loaded quantum dots engineered polymeric nanoparticles: Diagnosis/therapy combined approach. Eur. J. Pharm. Sci..

[34] Liu Z., Lin Q., Huang Q., Liu H., Bao C., Zhang W., Zhong X., Zhu L. (2011). Semiconductor quantum dots photosensitizing release of anticancer drug. Chem. Commun..

[35] Han C., Lalley J., Namboodiri D., Cromer K., Nadagouda M.N. (2016). Titanium dioxide-based antibacterial surfaces for water treatment. Curr. Opin. Chem. Eng..

[36] Haugen H., Lyngstadaas S. (2016). Antibacterial effects of titanium dioxide in wounds. Wound Healing Biomaterials.

[37] Kubacka A., Diez M.S., Rojo D., Bargiela R., Ciordia S., Zapico I., Albar J.P., Barbas C., Dos Santos V.A.M., Fernández-García M. (2014). Understanding the antimicrobial mechanism of TiO 2-based nanocomposite films in a pathogenic bacterium. Sci. Rep..

[38] Besinis A., De Peralta T., Handy R.D. (2014). The antibacterial effects of silver, titanium dioxide and silica dioxide nanoparticles compared to the dental disinfectant chlorhexidine on Streptococcus mutans using a suite of bioassays. Nanotoxicology.

[39] Karthikeyan G., Mohanraj K., Elango K.P., Girishkumar K. (2004). Synthesis, spectroscopic characterization and antibacterial activity of lanthanide–tetracycline complexes. Transit. Met. Chem..

[40] Peng P., Jiang W.-P., Li S.-M., Chen C.-Z., Liu G.-H. (2011). Synthesis, characterization and antibacterial activity of complex for gadolinium iodide with thiourea. Appl. Chem. Ind..

[41] Pradhan N., Peng X. (2007). Efficient and Color-Tunable Mn-Doped ZnSe Nanocrystal Emitters:  Control of Optical Performance via Greener Synthetic Chemistry. J. Am. Chem. Soc..

[42] Moulick A., Blazkova I., Milosavljevic V., Fohlerova Z., Hubalek J., Kopel P., Vaculovicova M., Adam V., Kizek R. (2015). Application of CdTe/ZnSe Quantum Dots in In Vitro Imaging of Chicken Tissue and Embryo. Photochem. Photobiol..

[43] Jelinkova P., Koudelkova Z., Milosavljevic V., Horky P., Kopel P., Adam V. (2016). Utilization of Selenium Nanoparticles with Schiff Base Chitosan as Antibacterial Agents. MendelNet.

[44] Herathge N.D.S., George J.T., Rowley D.A. (2011). Differential antimicrobial activities of Human Beta-Defensins against Methicillin Resistant (MRSA) and Methicillin sensitive (MSSA) *Staphylococcus aureus*. Science and Technology Against Microbial Pathogens.

[45] Mazumdar A., Haddad Y., Milosavljevic V., Michalkova H., Guran R., Bhowmick S., Moulick A. (2020). Peptide-Carbon Quantum Dots conjugate, Derived from Human Retinoic Acid Receptor Responder Protein 2, against Antibiotic-Resistant Gram Positive and Gram Negative Pathogenic Bacteria. Nanomaterials.

[46] Jelinkova P., Splichal Z., Jimenez A.M.J., Haddad Y., Mazumdar A., Sur V.P., Milosavljevic V., Kopel P., Buchtelova H., Guran R. (2018). Novel vancomycin-peptide conjugate as potent antibacterial agent against vancomycin-resistant *Staphylococcus aureus*. Infect. Drug Resist..

[47] Chudobova D., Cihalova K., Dostalova S., Ruttkay-Nedecky B., Merlos Rodrigo M.A., Tmejova K., Kopel P., Nejdl L., Kudr J., Gumulec J. (2014). Comparison of the effects of silver phosphate and selenium nanoparticles on *Staphylococcus aureus* growth reveals potential for selenium particles to prevent infection. FEMS Microbiol. Lett..

[48] Richter S.G., Elli D., Kim H.K., Hendrickx A.P.A., Sorg J.A., Schneewind O., Missiakas D. (2013). Small molecule inhibitor of lipoteichoic acid synthesis is an antibiotic for Gram-positive bacteria. Proc. Natl. Acad. Sci. USA.

[49] Vukomanovic M., Torrents E. (2019). High time resolution and high signal-to-noise monitoring of the bacterial growth kinetics in the presence of plasmonic nanoparticles. J. Nanobiotechnol..

[50] Stevenson K., McVey A.F., Clark I.B.N., Swain P.S., Pilizota T. (2016). General calibration of microbial growth in microplate readers. Sci. Rep..

[51] van Sorge N.M., Beasley F.C., Gusarov I., Gonzalez D.J., von Köckritz-Blickwede M., Anik S., Borkowski A.W., Dorrestein P.C., Nudler E., Nizet V. (2013). Methicillin-resistant *Staphylococcus aureus* Bacterial Nitric-oxide Synthase Affects Antibiotic Sensitivity and Skin Abscess Development. J. Biol. Chem..

[52] Schumacher A., Vranken T., Malhotra A., Arts J.J.C., Habibovic P. (2018). In vitro antimicrobial susceptibility testing methods: Agar dilution to 3D tissue-engineered models. Eur. J. Clin. Microbiol. Infect. Dis..

[53] Stasiak-Różańska L., Błażejak S., Gientka I. (2014). Effect of glycerol and dihydroxyacetone concentrations in the culture medium on the growth of acetic acid bacteria Gluconobacter oxydans ATCC 621. Eur. Food Res. Technol..

[54] Hogenkamp A., Herías M.V., Tooten P.C.J., Veldhuizen E.J.A., Haagsman H.P. (2007). Effects of surfactant protein D on growth, adhesion and epithelial invasion of intestinal Gram-negative bacteria. Mol. Immunol..

[55] Li R.C., Nix D.E., Schentag J.J. (1993). New turbidimetric assay for quantitation of viable bacterial densities. Antimicrob. Agents Chemother..

[56] Sacar M., Sacar S., Cevahir N., Onem G., Teke Z., Asan A., Turgut H., Adali F., Kaleli I., Susam I. (2010). Comparison of antimicrobial agents as therapy for experimental endocarditis: Caused by methicillin-resistant *Staphylococcus aureus*. Tex Heart Inst. J..

[57] Popova M., Martin C., Morgavi D.P. (2010). Improved protocol for high-quality Co-extraction of DNA and RNA from rumen digesta. Folia Microbiol..

[58] Welsh S., Peakman T., Sheard S., Almond R. (2017). Comparison of DNA quantification methodology used in the DNA extraction protocol for the UK Biobank cohort. BMC Genom..

[59] (2003). Quantification of DNA and RNA: A Spectrophotometric Method. eLS.

[60] Jorgez C.J., Dang D.D., Simpson J.L., Lewis D.E., Bischoff F.Z. (2006). Quantity versus quality: Optimal methods for cell-free DNA isolation from plasma of pregnant women. Genet. Med..

[61] Wilfinger W.W., Mackey K., Chomczynski P. (1997). Effect of pH and Ionic Strength on the Spectrophotometric Assessment of Nucleic Acid Purity. BioTechniques.

[62] Heger Z., Merlos Rodrigo M.A., Michalek P., Polanska H., Masarik M., Vit V., Plevova M., Pacik D., Eckschlager T., Stiborova M. (2016). Sarcosine Up-Regulates Expression of Genes Involved in Cell Cycle Progression of Metastatic Models of Prostate Cancer. PLoS ONE.

[63] Trivedi M.K., Branton A., Trivedi D., Nayak G., Bairwa K., Jana S. (2015). Spectroscopic Characterization of Disodium Hydrogen Orthophosphate and Sodium Nitrate after Biofield Treatment. J. Chromatogr. Sep. Tech..

[64] Perrin F.X., Nguyen V., Vernet J.L. (2003). FT-IR Spectroscopy of Acid-Modified Titanium Alkoxides: Investigations on the Nature of Carboxylate Coordination and Degree of Complexation. J. Sol-Gel Sci. Technol..

[65] Sondi I., Salopek-Sondi B. (2004). Silver nanoparticles as antimicrobial agent: A case study on E. coli as a model for Gram-negative bacteria. J. Colloid Interface Sci..

[66] Diana P., Sigifredo O., Jaime R., María Claudia V., Julieta P., Orville H., Jinnethe R., César A.A. (2002). First Characterization of a Cluster of VanA-Type Glycopeptide-Resistant *Enterococcus faecium* Colombia. Emerg. Infect. Dis. J..

[67] Qureshi N.K., Yin S., Boyle-Vavra S. (2014). The role of the Staphylococcal VraTSR regulatory system on vancomycin resistance and vanA operon expression in vancomycin-resistant *Staphylococcus aureus*. PLoS ONE.

[68] Liu B., Cheng W., Rotenberg S.A., Mirkin M.V. (2001). Scanning electrochemical microscopy of living cells: Part 2. Imaging redox and acid/basic reactivities. J. Electroanal. Chem..

[69] Bard A.J., Mirkin M.V. (2012). Scanning Electrochemical Microscopy.

[70] Kim S.R., Park M.J., Lee M.K., Sung S.H., Park E.J., Kim J., Kim S.Y., Oh T.H., Markelonis G.J., Kim Y.C. (2002). Flavonoids of Inula britannica protect cultured cortical cells from necrotic cell death induced by glutamate. Free Radic. Biol. Med..

[71] Shoemaker M., Cohen I., Campbell M. (2004). Reduction of MTT by aqueous herbal extracts in the absence of cells. J. Ethnopharmacol..

