# Supplementary material for: A Novel Biocompatible Titanium–Gadolinium Quantum Dot as a Bacterial Detecting Agent with High Antibacterial Activity
Source: Nanomaterials (Basel). 2020 Apr 17;10(4):778. doi: 10.3390/nano10040778 (PMC7221622; doi:10.3390/nano10040778)
Supplement: Supplementary file 1 [file nanomaterials-10-00778-s001.pdf]

Supplementary Material

A Novel Biocompatible Titanium–Gadolinium Quantum Dot as a Bacterial Detecting Agent with High Antibacterial Activity

Vishma Pratap Sur [1,2,*,†], Aninda Mazumdar [1,2,†], Amirmansoor Ashrafi [1,2], Atripan Mukherjee [1,2], Vedran Milosavljevic [1,2], Hana Michalkova [1], Pavel Kopel [3], Lukáš Richtera [1,2] and Amitava Moulick [1,2,*]

1 Department of Chemistry and Biochemistry, Faculty of AgriSciences, Mendel University in Brno, Zemedelska 1/1665, 61300 Brno, Czech Republic; xmazumda@mendelu.cz (A.M.); amirmansoor.ashrafi@mendelu.cz (A.A.); atripan@gmail.com (A.M.); grizlidripac@gmail.com (V.M.); hanabuchtelova@gmail.com (H.M.); oliver@centrum.cz (L.R.)

2 Central European Institute of Technology (CEITEC), Brno University of Technology, Purkynova 123, 62100 Brno-Královo Pole, Czech Republic

3 Department of Inorganic Chemistry, Faculty of Science, Palacky University, 17. listopadu 12, CZ-771 46 Olomouc, Czech Republic; paulko@centrum.cz

* Correspondence: vishmapratapsur@gmail.com or xsur@mendelu.cz (V.P.S); amitava.moulick@mendelu.cz or amitavamoulick@gmail.com (A.M); Tel.: +420-545-133-350 (V.P.S.); +420-545-133-350 (A.M.)

† These authors contributed equally to this work.

Received: 18 March 2020; Accepted: 16 April 2020; Published: 17 April 2020

1. FTIR analysis of TGQDs

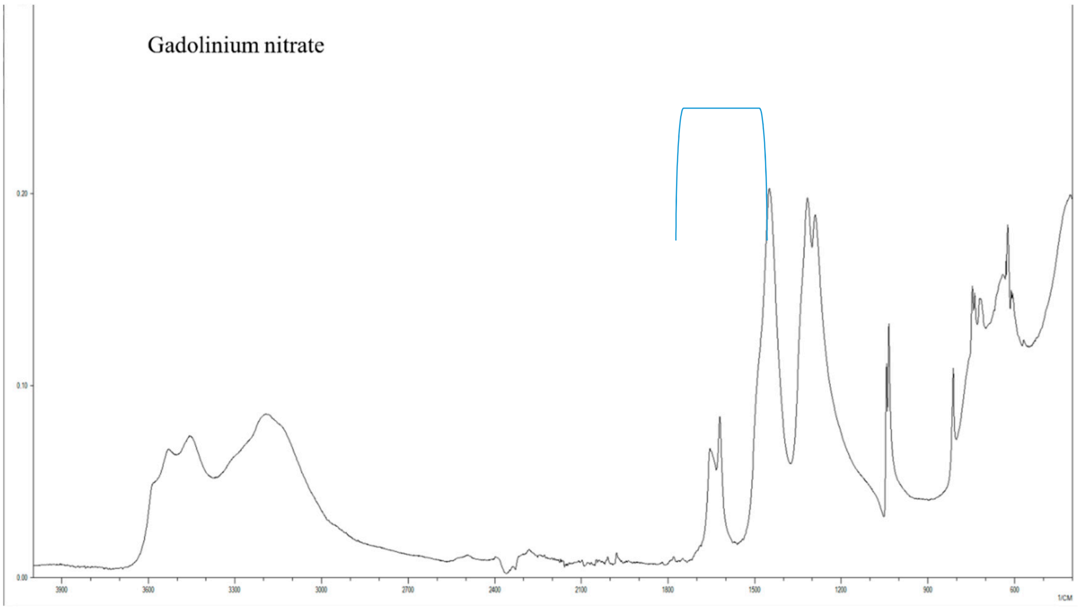

Figure S1. FTIR spectrum for Gadolinium Nitrate.

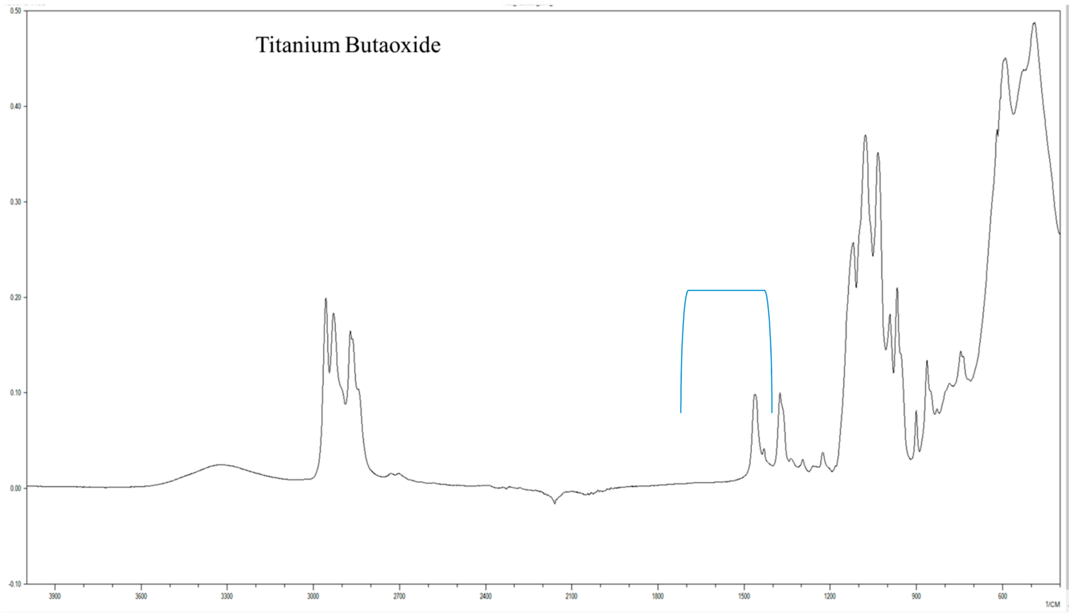

FigureS2. FTIR spectrum for Titanium Butoxide.

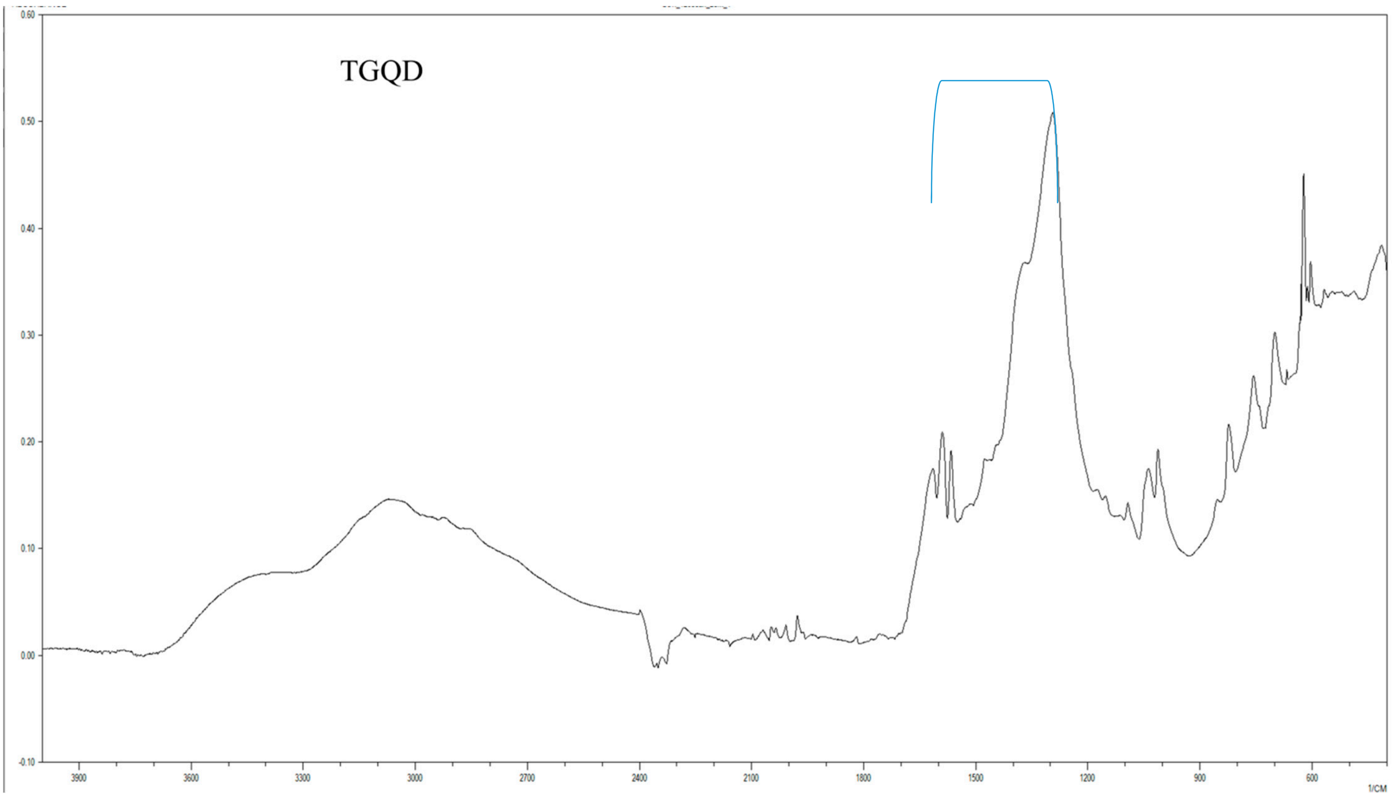

Figure S3. FTIR spectrum for synthesized TGQD.

2. Bacterial detection by absorbance

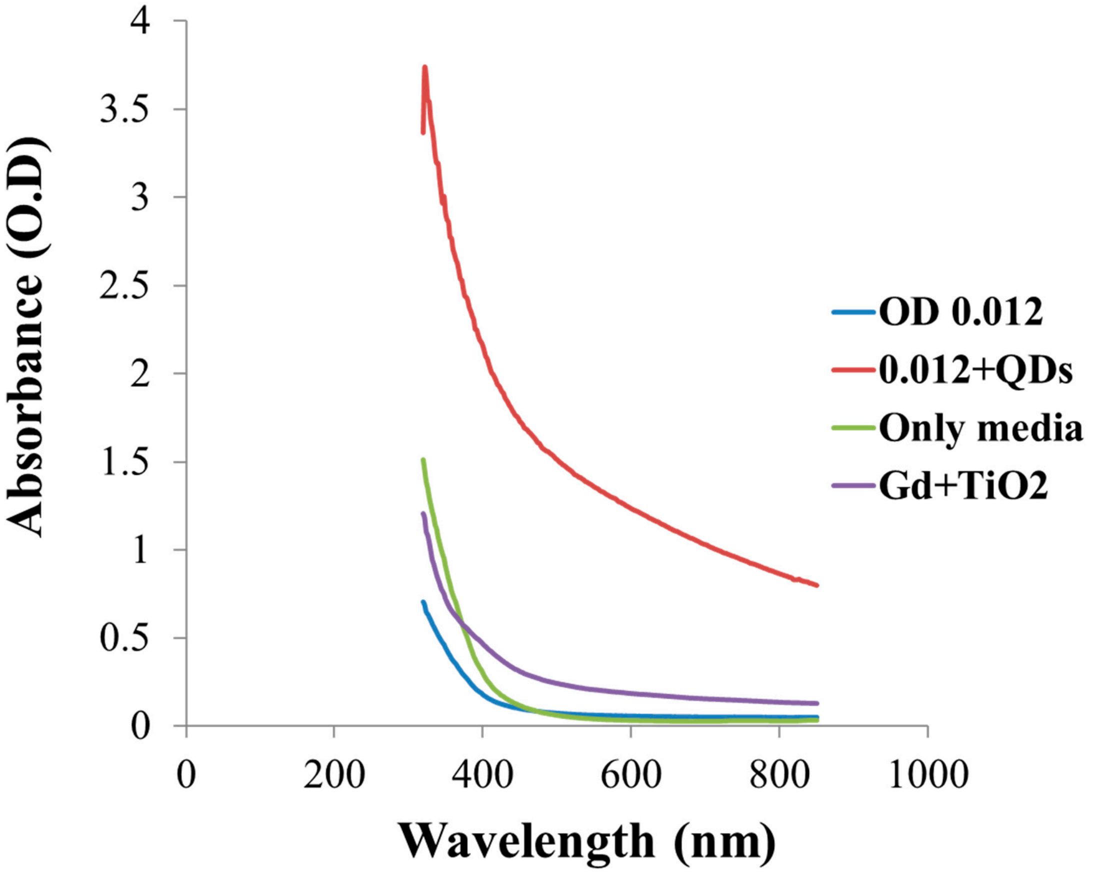

FigureS4. Bacterial detection by measuring absorbance spectra in presence of TGQD.

3. **Bacterial cell and Human cell staining**

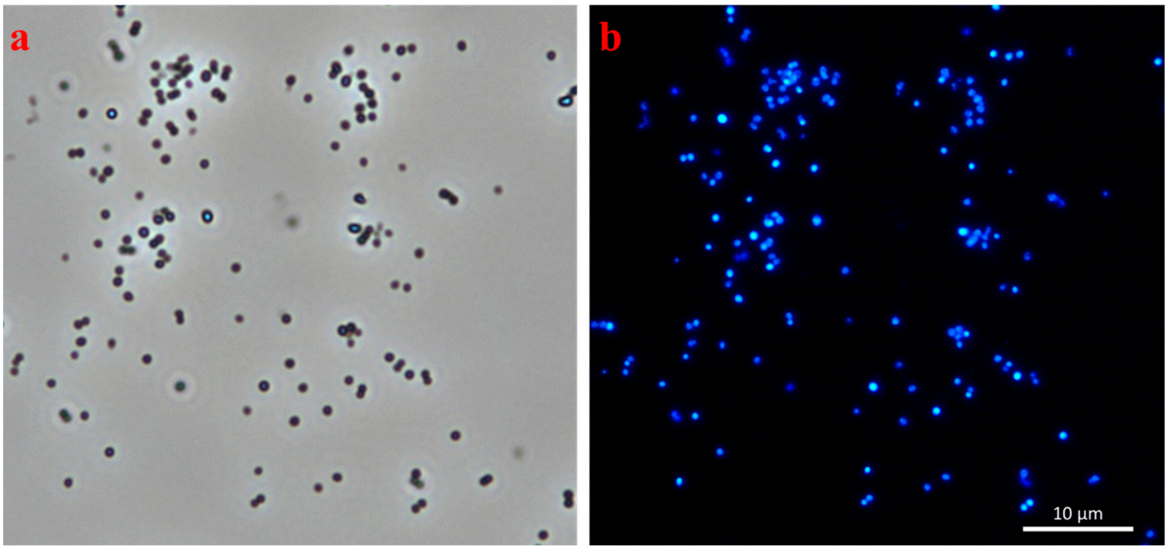

Figure S5. Bacterial cells stained with TGQD. (**a**) Phase contrast optical microscopic image of VRSA; (**b**) TGQD stained VRSA under fluorescence microscopic UV filter.

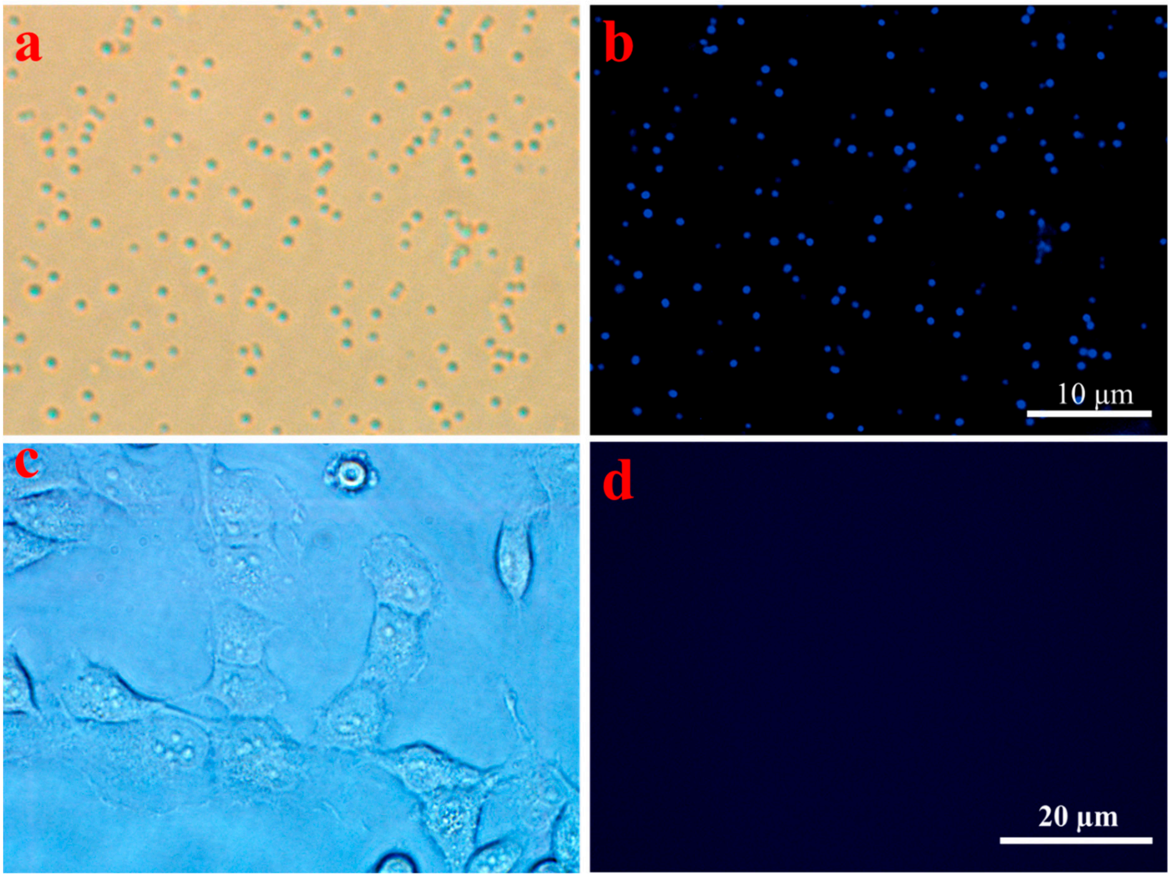

Figure S6. Bacterial cells and Human PNT1A cells were stained with SYTO9 and TGQD. optical microscopy image of bacterial cells (**a**); same field with TGQD staining (**b**); optical microscopy image of PNT1A cells (**c**). (**d**); PNT1A cells incubated with TGQD however TGQD did not stain the PNT1A cells (d).

4. Cytotoxicity of TGQDs

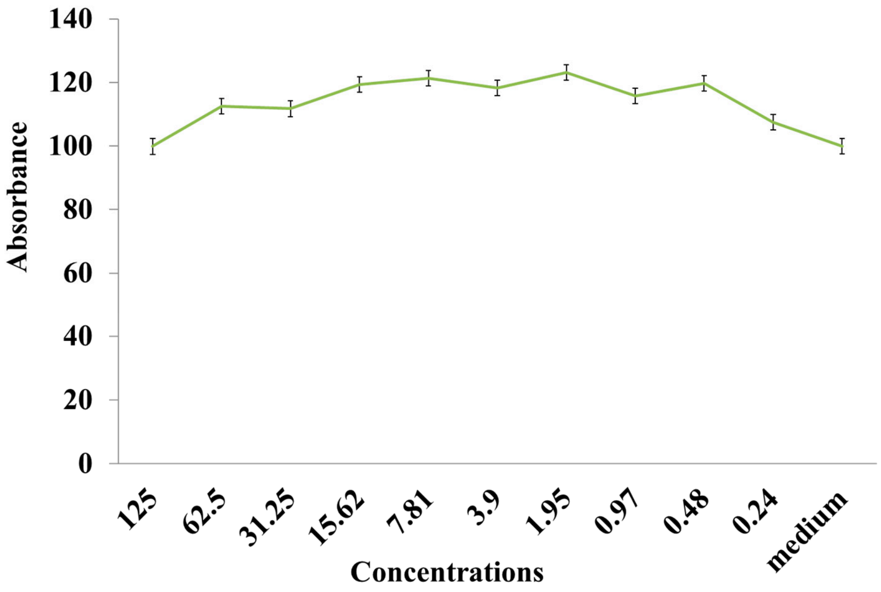

Figure S7. MTT assay against HBL-100 cell line.

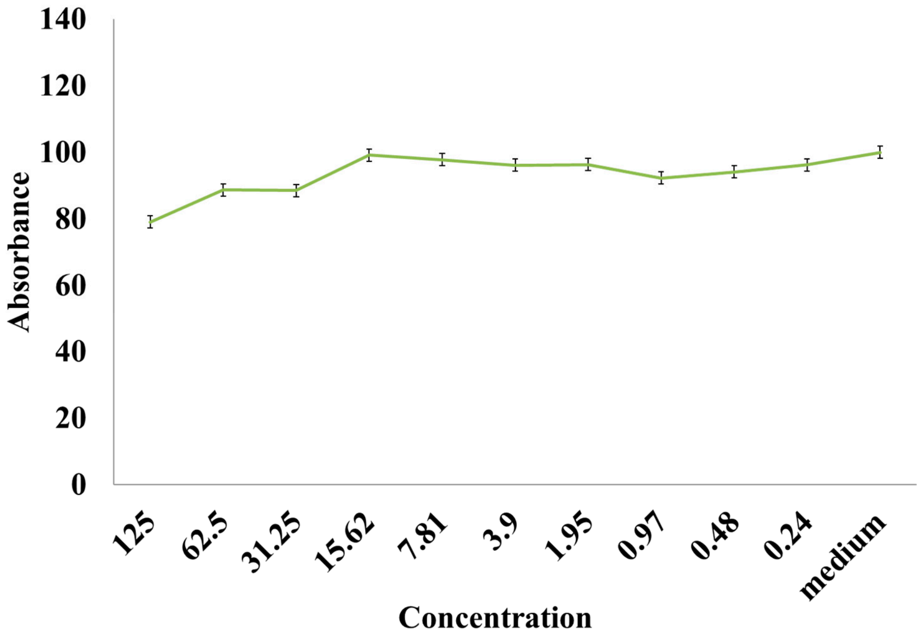

Figure S8. MTT assay against MDA-MB-231 cell line.
